# Parental Distress and Perception of Children’s Executive Functioning after the First COVID-19 Lockdown in Italy

**DOI:** 10.3390/jcm10184170

**Published:** 2021-09-15

**Authors:** Concetta Polizzi, Sofia Burgio, Gioacchino Lavanco, Marianna Alesi

**Affiliations:** Department of Psychology, Educational Science and Human Movement, University of Palermo, 90128 Palermo, Italy; concetta.polizzi@unipa.it (C.P.); gioacchino.lavanco@unipa.it (G.L.); marianna.alesi@unipa.it (M.A.)

**Keywords:** parental distress, COVID-19, children, executive functioning

## Abstract

The spread of the novel coronavirus (COVID-19), and the consequential first italian lockdown to minimize viral transmission, have resulted in many significant changes in the every-day lives of families, with an increased risk of parental burnout. This study explores the impact of the first COVID-19 lockdown in Italy on parental distress and parental perceptions of children’s executive functions (EFs). Participants were 308 Italian parents with children between 4 and 17 years of age; they were recruited through online advertisements on websites and social media, and they were given an online survey. The measures were: the balance between risks and resources (BR2) and the executive functioning self-report (EF). Findings of the study suggest that the most distressed parents perceived their children as less competent in EF, highlighting a cognitive fragility on attention, memory, and self-regulation (Pearson correlation coefficient, *p* < 0.05); significant differences were found between parents of children exhibiting typical and atypical patterns of development (ANOVA, *p* < 0.05). The study reinforces the need to provide families with psychological aid to support parental competence in restrictive lockdown conditions.

## 1. Introduction

From 10 March to 17 May, 2020, the Italian government imposed a national lockdown to limit viral transmission of COVID-19. This period, lasting about 70 days, was characterized by severe social restriction measures, including domestic confinement, working from home, closure of non-essential businesses, and closure of all schools with distance learning. Immediate consequences were vast, i.e., the loss of freedom, the separation of elderly relatives from contradictory information surplus, the uncertainty about COVID-19 infection and health, and the increasing use of social networks. Other additional conditions included the lack of personal space at home and family uncertainty, concerning economic status or inadequate supplies (food, drugs, and safety devices) [1,2,3,4]. Following a break-down of borders between work and home, parents faced several pressing concerns, rearranging their work/children’s schedules, as well as their children’s education routines, because of school closures. In a complementary way, children were asked to re-arrange their routines by giving up all out-of-home and after-school activities, ranging from sports/recreational activities to social relationships with peers [5,6].

All of these factors, in addition to concerns about specific outbreaks, i.e., due to fears about COVID-19 and the use of measures to prevent it, acted as psychological stressors and accounted for detrimental effects on the global well-being and mental health of both adults and children [7]. Moreover, during the home confinement, stressful effects were intensified by “distress contagion” among family members through spillover or crossover mechanisms. The spillover mechanism refers to the impact in which exposure to distress in one area can have a ripple effect (i.e., in personal functioning) in another area (e.g., from work or economic concerns to parenting skills), whilst the crossover mechanism refers to contagion from one member of the family to other family members [6].

Research on the psychological effects from previous epidemics and pandemics (e.g., SARS, MERS, H1N1, Ebola, equine influenza) corroborated with high rates of family distress, ranging from 30% in children to 25% in parents [8]. In particular, following a lockdown, to contrast severe acute respiratory syndrome (SARS) and Middle-East RESPIRATORY SYNDROME (MERS), psychological (anxiety, depression, irritability, anger, emotional exhaustion, etc.) and behavioral (hyperactivity, sleep disorders, angry, conduct problems, externalizing problems, withdrawn, or clingy…) symptoms were reported [9,10,11,12].

Furthermore, it has been well documented how a long breakdown could enhance psychological distress, with a progressive appearance of despair during the first 9 days, anxiety from the 15th to the 19th day, and anger from the 20th to the 31st day, until a condition comparable to post-traumatic distress disorder (PTSD) occurs [10,13].

In a large Chinese survey involving 52,730 participants, the rate of psychological distress due to the COVID-19 outbreak was about 35%, with gender and age differences. Higher levels of distress were found in the female group and age range between 18 and 30 years or above 60 years [14]. An online survey carried out in Italy with 2766 respondents showed increased levels of frequency of anxiety, depression, and distress compared to the European epidemiological statistics. More in depth, higher levels of distress were reported by women, higher levels of depression were showed by individuals with a previous history of trauma or medical diseases, or having an acquaintance infected with COVID-19, higher levels of anxiety occurred in younger individuals and people who had an infected relative [15]. Of particular interest was the association between depression and the condition characterized by not having children. This is coherent with findings that highlighted how being a parent or living with children is a factor that enhances psychological distress during a lockdown or quarantine [16].

In particular, high psychological stress levels have been found in parents of children with special needs and exhibiting atypical patterns of development, due to a disability or a chronic medical condition [17,18,19]. Several studies pointed out the high prevalence of depressive symptoms and significant changes in strain observed among parents of special needs children during the COVID-19 outbreak; they demanded greater attention from mental health practitioners and rehabilitation care providers [20].

In light of the pandemic effects on psychological health, in particular for parents [13,21,22] during this critical period, there was an increase in requests for psychological support [4,23]. To respond to this emerging need, there has been a global proliferation of online psychological support services; these services were often free and promoted by government authorities, such as the Italian Ministry of Health [24]. In Italy, among the adults who asked for help from these services, many parents reported great difficulty in managing the relationships with their children, especially if they had some evolutionary fragility or special needs [24].

Almost all studies that analyzed the psychological conditions of parents indicated that specialist responses (psychological and psychotherapeutic) are fundamental for the support needs expressed by parents [25,26]. In other words, many parents during the pandemic needed the support of mental health specialists to cope with difficulties.

Higher levels of distress, negative emotions, such as anger or frustration, and negative cognitions, such as pessimism or perfectionism, were displayed by parents with personality profiles characterized by high neuroticism, with patterns of worrying, emotional variability, and feelings of insecurity [13].

In turn, parental distress negatively influenced the caregiver’s ability to manage their children at home and to adopt proper disciplinary measures [13].

Petrocchi et al. [27] found that mothers with higher distress levels attributed more negative emotions to their children, with consequences on children’s adaptive functioning [3].

In regard to the children, as found by the first studies carried out in China, behavioral problems due to COVID-19 lockdowns, ranged from 4.7% to 10.3% in childhood [28].

Psychological consequences varied from anxiety to depression, regression symptoms, panic attacks, irritability, restlessness, and feelings of loneliness [3,29,30,31,32]. Indeed, vulnerability factors, such as developmental age, educational, and socioeconomic status, previous special needs, or mental health disease, seemed to moderate the appearance of psychological problems [33].

Clinginess and the fear of contagion were more frequent in younger children aged, approximately, 3 to 6 years; inattention and inquiry persistence were more frequent in older children aged 6 to 18 years [34].

However, despite the increasing research on parental distress effects, to the best of our knowledge, minor research has been produced to investigate the interplay between parental distress and children’s executive functions (EF) during a lockdown period.

Executive functioning (EF) is a set of core cognitive processes for development that consist of a variety of higher-order cognitive processes; it plays a key role in mental and physical health. Moreover, EF is involved in a wide range of “long-life” aspects, ranging from school readiness to job success, marital harmony, and public safety [35].

Executive functions involve a network of frontal-parietal areas, are greatly heritable, and are supposed to be polygenic. Three main components were identified as being potentially important: inhibition, switching, and working memory [36]. Inhibition is the ability to control and repress a prevailing response in support of another response or no response. Switching is the ability to switch from one task or mindset to another. Finally, working memory is the capacity to monitor and manipulate items and mental representations in the mind [35]. These three components are theoretically described as a model of “unity and diversity” because they refer to distinct but inter-correlated cognitive functions. As demonstrated by structural equation modeling, the three functions contribute in a different way to allow a successful performance on tasks, tapping memory, inhibition, fluency, and attentional shifting; therefore, the need to recognize the unity and diversity of executive functions is suggested [37].

Moreover, these main components are closely related to other processes, such as fluency and planning. Fluency denotes the ability to produce, in a given amount of time, as many items as possible (e.g., words, colors, etc.), and planning is the ability to identify, employ, and monitor a sequence of thoughts or actions to achieve a specific goal [38].

An inadequate family context, lacking both material and psychosocial supports, is considered to be a risk condition that hampers a child’s development of EF [39,40]. Family functioning explains up to 20% of the variance of a child’s performance in working memory and EF tasks [41].

Following an ecological perspective, previous research demonstrated that EFs, for their long development, are very sensitive to macro-contextual (e.g., cultural contexts or SES) and micro-contextual (e.g., family members psychological variables, language, parent-child relationships, parenting skills) factors [42]. Adverse environments characterized by high levels of parental distress account for increasing parental control strategies and limit the use of scaffolding measures to support the gradual increase of children’s autonomy in decision-making processes, behavioral, and emotional management, and the use of strategies to cope with distress events. The extreme consequence could be an impairment or delay in long-term EF development [43]. Children with poorer EFs had a higher level of salivary cortisol, which is the distress hormone [44]. Moreover, parents’ mental health factors, such as acute distress, depression, and anxiety, can impair parenting skills and children’s later development of working memory, inhibition, and cognitive flexibility [43,45,46]. Parents with experiences of daily distress and worries about life goals can feel less connected to their children, engage less in cognitively challenging tasks, produce a distressful or chaotic family context and, as a consequence, can have behavior and parent–infant interaction that obstructs the development of a child’s self-regulatory skills, which ends up influencing cognitive development [47,48,49]. On the other hand, a lack (or a break) from school learning is a risk factor for the increased distress of a child, reduced inhibitory control, and cognitive flexibility, as well as concerns surrounding planning, attention, or decision making [50]. Contemporary research has documented how, during the lockdown, children’s self-regulation capacities and inhibitory controls seem to have been negatively influenced by an increase of the mother’s negative emotions, by changes in the mother’s sleep quality and in the perception of time, by the breakdown of daily routines and after-schools habits [51]. Furthermore, attention deficit hyperactivity disorder (ADHD) symptoms in children worsened following the lack of daily routines, extra-familiar social relationships, and the increase of family distress [12].

In light of the literature reviewed above, the present study assessed the relationships between parental distress and children’s EF, as perceived and reported by their parents. In regard to parental distress, this study assumed the balances between risks and resources theory [52,53] that explains parental distress as a condition of parental burnout, resulting from an imbalance between parental risks and protective factors. Parental burnout develops when parental resources are insufficient to meet the demands/risk factors that significantly increase parental distress levels (e.g., parental low emotional intelligence, perfectionism, poor childrearing practices, lack of support from the co-parent, lack of social support).

An online cross-sectional survey was performed to measure the above-mentioned variables immediately after the end of the first spring Italian lockdown (from March to May 2020) and prior to the following autumn (partial) lockdown (since October 2020). This period was selected because critical questions remain to be answered about the short-term effects of pandemic events on cognitive development whilst pandemic long-term effects on mental health have been well-documented in previous pandemic conditions.

Given these concerns the following goals were investigated:

Goal 1: parental distress following first COVID-19 lockdown would be influenced by parental variables (age, education, job or working condition, couples’ conditions, support needs during the lockdown) and the child’s typical/atypical patterns of development.

Goal 2: parental perception of children’s EF would be influenced by specific parental variables (age, education, job or working condition, couples’ conditions, support needs during the lockdown), and the child’s typical/atypical patterns of development.

Goal 3: parental distress following the first COVID-19 lockdown would be related to the perception of children’s EF. Higher levels of parental distress would be associated with their perception of worse EFs in their children.

## 2. Materials and Methods

### 2.1. Participants

Participants were recruited through online advertisements on websites and social media (Facebook and WhatsApp).

Inclusion criteria were: (a) being a parent; (b) being at least 18 years old, and (c) having a child between 4 and 17 years of age.

The collected data refer to a sample of 308 parents, 278 mothers (90.3%), and 30 fathers (9.7%). Most parents were aged between 36 and 45 years (52.9%) and were of Italian nationality (98.7%). The parents were mostly married (84.4%); an equal number were either separated with joint custody (and who had the child at home during the lockdown) (7.1%) or cohabiting (7.5%). Only 1% of parents were single.

A degree was the most popular qualification (39.6%), followed by a high school diploma (30.5%). A minority of participants had a Ph.D. or a specialization (15.6%), a professional diploma (7.8%), a primary school certificate, or a middle school diploma (6.4%).

A total of 35.1% of the participants were government employees, while 19.2% were freelancers, 14.3% were teachers, and 10.7% were housewives. Other professions were practiced, but in very low percentages. Moreover, most of these occupations were carried out in smart working modes (45.5%) during the first lockdown caused by the COVID-19 pandemic, 20.8% of the sample declared that they continued to work at the workplace, while a small number of parents said they did not work due to a suspension of their activities (12.7%), because they were temporary layoffs (3.9%), or because they lost their jobs (3.2%).

The children of these parents were mostly boys (57.8%) while 42.2% were girls. The children taken into consideration were mostly first-born (67.9%), followed by the second-born (25.3%), and finally by the third-born and beyond (6.8%). The first-born considered by the parents for the study were mostly between the ages of 11 and 13 (41.6%), followed by children between the ages of 7 and 10 (30.7%) and, finally, children between the ages of 4 and 6 years (27.7%); neither parent involved in the study had sons between the ages of 14 and 17 years old. Furthermore, most of the 11–13-year-old and 7–10-year-old first born children had at least one sibling.

Concerning the developmental conditions of the children, 270 were children exhibiting “typical” patterns of development and 38 were children exhibiting atypical patterns of development, which included learning disabilities, ADHD, genetic syndrome, intellectual disabilities, sensorial disabilities, autism spectrum disorder, or chronic or severe pathologies.

Neither parent was infected with COVID-19 during the research.

### 2.2. Measures and Procedure

An online survey was administered from June to October 2020. Participants were recruited through online advertisements on websites and social media (Facebook and WhatsApp) via a snowball sampling strategy.

All participants were informed about the aims and procedures of the study by a brief description of the study; they gave their informed consent (via the survey) before filling out the survey. Participation was voluntary and anonymous. The survey took about 20–25 min to complete.

The study was conducted in accordance with the Declaration of Helsinki and was approved by the Bioethics Committee of the University of Palermo (no. 13/2020).

Collected parental sociodemographic data were: gender, age, nationality, region, marital status, level of education, work regimen before and during COVID-19 pandemic, habits before and during the COVID-19 pandemic, social relations after the COVID-19 emergency, the use of social networks, psychological support needs during the COVID-19 pandemic, discomfort during the COVID-19 emergency, and ideas relating to the process of the pandemic. Concerning the children, data included gender, birth order, pathologies, or disabilities.

Parental distress was measured by the balance between risks and resources (BR2; [52]). It was a self-report questionnaire. Specifically, the BR2 instrument reliably measured parental balance between risks (parental distress-enhancing factors) and resources (parental distress alleviating factors). This tool was composed of 39 bipolar items, in which parents were asked to read carefully each sentence and express their degree of agreement, using a 10-point answer scale of values ranging from −5 to +5. Of these, 14 items defined common antecedents as risk factors, indicating predictors of job and parental burnout (e.g., “It is difficult for me to reconcile my family life and my professional life”) and 25 items defined specific antecedents showing aspects strictly related to parental burnout (e.g., “Due to my parenting responsibilities, I can never find time for myself”). The total score ranged between −195 and +195. A positive score revealed the prevalence of parental resources, whereas a negative score indicated the risk of parental burnout; the “0” scores revealed equal levels of risks and resources. The original version was translated and adapted to the Italian context with the author’s permission. The original administration procedure was used. The results of the EFA showed that the principal component analysis identified two factors that explained the 47.21% variance. The mean sampling adequacy (Bartlett’s test) was 6889.42 (*p* < 0.001) and the Kaiser–Meyer–Olkin (KMO) was 0.917.

The internal consistency was verified through the Cronbach’s Alpha test, given that the Cronbach’s Alpha reliability is considered within an acceptable range, of around 0.70. The reliability values were of α = 0.96 for the global scale, α = 0.89 for the common antecedents subscale, and α = 0.94 for the specific antecedents subscale.

The parental perception of children’s EF was measured through the executive functioning self-report (EF-SR) [54]. This was composed of 20 items, conceptualizing, as a system of perceptions, about their child’s cognitive abilities in the management of environmental conditions, such as working memory (e.g., “My child is not good at remembering sequences of items, for example, numbers or words”), attention (e.g., “My child has difficulty ignoring extraneous thoughts when he/she performs a task”), shifting (e.g., “My child has difficulty moving from one task to another (for example from a math task to a science task”), planning (e.g., “My child has troubles performing tasks that have more steps”) and inhibitory control (e.g., “My child has difficulty with concentration while working in the classroom”). All items used a four-point Likert scale ranging from 1 (always) to 4 (never) to quantify the frequency of use. Higher scores indicated minor difficulties on EF tasks.

A total score (from 20 to 80) and sub-scores (from 4 to 16) for each area were obtained. The reliability values were of α = 0.95 for the global scale, α = 0.87 for the working memory subscale, α = 0.80 for the attention subscale, α = 0.86 for the shifting subscale, α = 0.76 for the planning subscale, α = 0.79 for the inhibitory control subscale.

### 2.3. Statistical Analysis

In regard to goals 1 and 2 of the study, analyses of variance (ANOVA) were run through the Statistical Program for Social Sciences (SPSS) (IBM SPSS Statistics for Windows, Version 24.0. IBM Corp., Armonk, NY, USA) to investigate parental distress and parental perception of children EFs as a function of independent variables, such as parental and children’s variables; the independent variables were parental characteristics (age, education, job, working condition during the lockdown, couples’ conditions, psychological support needs) as well as children’s characteristics (age, order of birth and typical/atypical patterns of development) whilst the dependent variables were the scores on BR2 subscales (global scale, common antecedents subscale and specific antecedents subscale) and EF-SR questionnaire subscales, respectively (total EF, working memory, inhibition, shifting, planning and attentional control). For the analysis of variance, the parental gender variable was not considered, because the sample consisted of 90.3% mothers. The partial eta square was used to estimate effect size, and in according with Cohen (1988), was interpreted as small (0.01 < η^2^_p_ ≤0.06), medium (0.06 < η^2^_p_ ≤ 0.14), and large (η^2^_p_ > 0.14). Moreover, in about one-third of the study, correlation analyses were performed by Pearson’s correlation coefficient, to assess the association between parental distress and parental perception of children’s EF; subsequently, considering the correlational data, a linear regression analysis was performed with the stepwise method.

## 3. Results

### 3.1. Parental Distress

Parental distress descriptive data are showed in Table 1, Table 2, Table 3, Table 4, Table 5 and Table 6.

Concerning parental distress, the global distress (F(1, 308) = 4981; *p* = 0.02; η^2^_p_ = 0.02), specific antecedents (F(1, 308) = 3.748; *p* = 0.05; η^2^_p_ = 0.01), and common antecedents (F (1, 308) = 6.225; *p* = 0.01; η^2^_p_ = 0.02) differed in a significant way between parents having psychological support needs and parents not having this support, in spite of the low effect size value. The higher level of distress on all three scales were obtained by parents who required and obtained psychological or social support.

There were also significant group differences on global distress (F (1, 308) = 8.955; *p* < 0.01; η^2^_p_ = 0.03), common antecedents (F (1, 308) = 12.063; *p* = 0.001; η^2^_p_ = 0.04), and specific antecedents (F (1, 308) = 6.383; *p* < 0.01; η^2^_p_ = 0.02) between parents of children exhibiting “typical” patterns of development and parents of children exhibiting atypical patterns of development, in spite of the low effect size value. Parents having children with special needs reported higher rates of distress on all scales.

### 3.2. Parental Perception of Children’s Executive Functions and Correlations with Parental Distress

The parental perception of children’s EFs descriptive data are given in Table 7, Table 8, Table 9, Table 10, Table 11 and Table 12; while correlation data are showed in Table 13.

Concerning the parental perception of children’s EFs, statistically significant differences were found as the effect of having children with typical/atypical patterns of development, parental age, parental educational level, and children’s age; in particular, there was a significant effect on having children with typical/atypical patterns of development on working memory (F(1, 307) = 4.24; *p* < 0.05; η^2^_p_ = 0.014), attention (F(1, 307) = 5.271; *p* < 0.01; η^2^_p_ = 0.017) and shifting (F(1, 307) = 3.806; *p* < 0.05; η^2^_p_ = 0.012), in spite of the low effect size value. Parents of children exhibiting atypical patterns of development rated the lowest performance of their children on working memory, attention, and shifting tasks. Moreover, there was a significant effect of parental age on children’s inhibition (F(1, 307) = 3.87; *p* < 0.01; η^2^_p_ = 0.02); in fact, younger parents (<36 years old) rated the lowest performance of their children on inhibitory control tasks. Furthermore, there was a significant effect of children’s age on parental perception of working memory (F(1, 307) = 3.491; *p* < 0.05; η^2^_p_ = 0.02), planning (F(1, 307) = 5.188; *p* < 0.01; η^2^_p_ = 0.03), inhibition (F(1, 307)= 6.926; *p* = 0.001; η^2^_p_ = 0.04), and EF total score (F(1, 307) = 4.387; *p* = 0.01; η^2^_p_ = 0.03); in particular, the younger children (4–6 years) were perceived by their parents as less able to cope with tasks of working memory, planning, and inhibition. Moreover, there was a significant effect of the parents’ educational levels on parental perception of children’s EFs inhibition (F(1, 307) = 2.646; *p* < 0.05; η^2^_p_ = 0.03).

Finally, correlation analyses were performed to assess the association between parental distress and parental perception of children’s EFs. Positive significant correlations (*p* < 0.01) were found among each component of parental distress and each component of the child’s EF perception, demonstrating how parents with higher levels of resources and minor levels of distress perceived their children as more able to perform tasks requiring EF competencies during a COVID-19 lockdown.

Considering the correlational data, a multiple linear regression was performed with the stepwise method (see Table 14); the variable “common antecedents” was removed in relation to all pf the EF components, as a probability of F ≥ 0.100 was found (significance of F with regard to the common antecedents: 0.989 for working memory, 0.929 for attention control, 0.269 for planning, 0.874 for shifting, and 0.157 for inhibition). The data are in line with what we expect, based on the correlations carried out, which showed a stronger correlation between the different EFs considered and the specific antecedents.

## 4. Discussion

The current study provided a multifaceted investigation of parental distress and parental perception of children’s EFs following the spread of the novel coronavirus (COVID-19) pandemic and the first lockdown in Italy.

Based on previous data demonstrating how distress affected parents more than those who did not have children, as well as younger parents [55,56], we hypothesized that, during the first COVID-19 lockdown, the parents who experienced the greatest distress perceived their children as more difficult to manage and more at risk of developmental frailty. More distressed parents, indicating a lack of resources or a prevalence of risk factors, are more likely to be too overwhelmed by the pandemic; this prevents them from supporting their children and responding to children’s questions, fears, and difficulties [57,58]. When children do not find emotional containment and responsive answers to their preoccupations from their parents, they are more likely to show higher levels of distress, with more difficulties in emotional and behavioral domains, such as inattention, concentration problems, and dysregulation [26,58].

Our findings confirm previous research by showing a general distressful condition in most of the parents who participated in this study [26,59,60]. This distressful condition is underlined by an average (and a widely variable) level of claimed resources; in fact, some parents (<36 years old) described their conditions by referring to the presence of moderate available resources, other parents (36–45 years old) indicated very few resources.

Furthermore, as previous studies showed [52,61,62], the risk factors for parenting distress are closely related to sociodemographic characteristics, such as child features and family functioning; our data on parental distress focused on the possible effects of sociodemographic variables, highlighting a high-risk group for parenting-related distress, characterized by the following factors: having a child with atypical patterns of development and needing specialist support (psychological or psychotherapeutic intervention) during the pandemic.

These data underline how, during the pandemic, the lack of important resources, such as adequate social support by family and friends, could be predictors of parental burnout (common antecedents). The lower rates of distress levels in parents who declared to have asked for help during the lockdown, such as professional help, underline the importance of psychological support being offered to parents in this phase of an epochal crisis. Moreover, being the parent of a child exhibiting atypical patterns of development was found to be a big source of distress, referring both to general predictors of parental burnout (common antecedents) and aspects strictly related to parental burnout (specific antecedents). This condition also affected parental perception of EF children. Children exhibiting atypical patterns of development were perceived as less able to manage and perform tasks requiring working memory, shifting, and attention processes during the lockdown. On the other hand, this is coherent with well-documented results in the literature showing a poorer performance on EF, especially working memory, in children with special needs [63].

According with some current research [64,65,66], parents of children with disabilities or chronic disease suffered the most from a complete lockdown, experiencing several new problems and increasing those already existing before the pandemic. A large amount of literature demonstrates how parents of children exhibiting atypical development experienced higher levels of parental distress compared to parents of children exhibiting typical patterns of development [17,18,19,67]; however, during the lockdown, they reached higher levels of distress, just because they started from disadvantaged conditions. Our study confirmed the negative effects of home confinement on parental distress when children have a disability or developmental fragility; the unexpected lifestyle changes generated by the COVID-19 pandemic, were even more difficult for children exhibiting atypical development, as well as for their families [68], especially because the professional support of those specialists (physicians, therapists, psychologists, etc.) who took care of children had decreased. Parents had to reorganize the daily activities and structure them according to their children’s needs, so this condition influenced both the wellbeing of parents and the psychological functioning and adjustment of the child [6,69,70]. Moreover, vulnerability factors, such as previous special needs, were demonstrated to enhance the appearance of psychological problems because of fears and worries concerning the worsening of atypical conditions, and the lack and/or limitation of external specialist support [33]. The literature has demonstrated how parents of children with disabilities are more likely to experience higher levels of parental distress characterized by perceptions of an imbalance between parenting requests and personal available resources. This can lead them to manage their children’s education, “less sensitively”, using less “efficacy-coping” strategies, or decreasing their ability to face challenging tasks with increasing risk of exacerbating their disability.

Therefore, we could hypothesize that, during the first COVID-19 lockdown, parents were more distressed compared to the stress faced during normal life conditions, and this might have increased their difficulty to manage the normal parental functions of caregiving and scaffolding [26]. Many parents likely experienced difficulties, in regard to satisfying the real needs of their children; at times, they might have overstimulated or hyper-controlled them, but often they hypo-stimulated them with little attention, in the absence of educational activities, in which the child could have otherwise experienced autonomy, self-efficacy, etc.

Moreover, our findings showed significant relationships between parental distress levels and the perception of the child’s cognitive abilities. The most distressed parents perceived their children as less competent in EFs, highlighting their cognitive fragility in attention, memory, and self-regulation tasks. However, the opposite direction of this relationship is admissible and plausible, given that correlation analyses do not allow establishing any causal link. Parents who perceive their children as less competent on EF tasks might have experienced more distressful requests of parenting and scaffolding to compensate for the absence of external specialist help during the lockdown [43,45,46]. Thus, these parents perceived themselves as having minor resources to face distressful events.

Although the main goal of this study was to observe short-term effects of pandemic events after the first strict lockdown, and establish a bivariate association between investigated variables, following the above-discussed correlational findings, linear regression analyses were carried out. These findings showed the higher predictive value of specific antecedents on all EF components, working memory, attention control, planning, shifting and inhibition. We can conjecture that the distress conditions strictly related to parental burnout make parents more impatient, less tolerant, and less able to manage their children at home, and to accept and support the developmental fragility of their children. Thus, these parents can adopt education measures and parent–child interactions that hamper their child’s self-regulatory skills, and end up influencing later cognitive development [47,48,49]. However, this causal relationship should be explored in more depth in the future; longitudinal studies can confirm these preliminary results.

## 5. Conclusions

To conclude, the findings of this study suggest that the first COVID-19 lockdown strongly influenced parental distress and their resources as well as parental perception of their children on working memory, attention, and shifting tasks, especially in the case of previous atypical development conditions. Thus, the evidence from this study reinforces the need to provide families with psychological aid, even in restrictive lockdown conditions, through different modalities, ranging from telephonic/electronic media platform consultations to online workshops that are able to support and/or enhance parenting skills, psychological techniques to deal with anxiety and parental distress, relaxation exercises, art therapy, and mindfulness training [6]. In the pandemic scenario, the role of psychological intervention is crucial for everyone, but it is needed for families of children with atypical development that experience temporary interruption of care assistance due to the increase in emotional fatigue (characterizing parenting strategies) and in children’s pre-existing vulnerabilities. Practitioners need to implement autonomy-supportive programs to teach parents how to cope with stress, with the indirect aim to optimize outcomes for children with special needs. For example, programs to decrease parental stress reinforce the use of task-oriented and emotion-oriented coping strategies, to deliver the satisfaction of parental competence, even in abnormal pandemic conditions.

The strength of this study is that it contributes toward bridging the gap in the lack of research on parental distress and children’s EF impairments during the COVID-19 lockdown, as affected by short-term consequences of pandemic conditions.

Nevertheless, future research is necessary to remedy the shortcomings of this study. The most important limitation lies in the relatively small size of our sample. This was because the recruitment of participants took place in a well-defined time-lapse, immediately after the end of the first spring lockdown in Italian, prior to the following autumn partial lockdown. This period was selected to investigate the short-term effects of pandemic events on cognitive development. Moreover, a low effect size for most comparisons was found, indicating the need of further analyses on other eventual variables, which play a role in the relationship between variables we investigated. Another shortcoming of this study lies in methodological concerns, as the measurement of children’s EFs is based not on the administration of cognitive performance-based tests, but rather on rating scales that measured children’s EFs through a parent-reported questionnaire, revealing parental perceptions of their children’s cognitive abilities. The choice of this measure was forced by the need to carry out research online, given the pandemic conditions. However, this questionnaire was found, by previous researchers, to be an ecologically valid indicator of EF functioning in concrete, complex, and everyday life situations, as well as suitable for inclusion in research projects with children who require the study of numerous variables.

## Figures and Tables

**Table 1:** Parental distress descriptive data and ANOVA analyses for parental psychological support needs and the presence of a child exhibiting atypical patterns of development.

	Psychological Support Needs M (SD)		Child Atypical Development M (SD)	
	No (*N* = 206)	Yes (*N* = 102)	No (*N* = 270)	Yes (*N* = 38)
TOT BR^2^ *	66.8 (63.6)	50.2 (57.1)	65.2 (60)	33.5 (68.6)
	F = 4.981; *p*-value = 0.026; η^2^_p_ = 0.02		F = 8.955; *p*-value = 0.003; η^2^_p_ = 0.03	
	Tot (*N* = 308) 61.3 (61.9)		Tot (*N* = 308) 61.3 (61.9)	
Common Antecedents **	20.8 (23.2)	13.9 (21.7)	20.2 (21.8)	6.6 (27.1)
	F = 6.225; *p*-value= 0.013; η^2^_p_ = 0.02		F = 12.063; *p*-value = 0.001; η^2^_p_ = 0.04	
	Tot (*N* = 308) 18.5 (22.9)		Tot (*N* = 308) 18.5 (22.9)	
Specific Antecedents ***	46 (42.9)	36.2 (38.9)	45 (41)	26.8 (44.5)
	F = 3.748; *p*-value = 0.054; η^2^_p_ = 0.01		F = 6.383; *p*-value = 0.012; η^2^_p_ = 0.02	
	Tot (*N* = 308) 42.7 (41.8)		Tot (*N* = 308) 42.7 (41.8)	

* Min and max scores: −195; +195. ** Min and max scores: −70; +70. *** Min and max scores: −125; +125.

**Table 2:** Parental distress descriptive data and ANOVA analyses for child age, birth order, and parental age.

	Child Age M (SD)			Order of Birth M (SD)			Parental Age M (SD)		
	4–6 (*N* = 70)	7–10 (*N* = 101)	11–13 (*N* = 137)	First Born (*N* = 209)	Second Born (*N* = 78)	Third Born and beyond (*N* = 21)	<36 (*N* = 50)	36–45 (*N* = 163)	>45 (*N* = 95)
TOT BR^2^ *	53.6 (58.9)	60 (59.9)	66.2 (64.8)	58.5 (64.4)	66.6 (59.9)	70.1 (54.2)	60.1 (60.4)	55.5 (61.8)	72 (62.1)
	F = 0.996; *p*-value = 0.370; η^2^_p_ =0.006			F = 0.719; *p*-value = 0.488; η^2^_p_ = 0.005			F = 2.156; *p*-value = 0.118; η^2^_p_ = 0.01		
	Tot (*N* = 308) 61.3 (61.9)								
Common Antecedents **	14.4 (20.4)	18.4 (22.4)	20.7 (24.3)	17.2 (23.5)	22.1 (22)	18.6 (18.8)	17.7 (20.9)	16.2 (22.8)	23 (23.6)
	F = 1.781; *p*-value = 0.170; η^2^_p_ = 0.01			F = 1.305; *p*-value = 0.273; η^2^_p_ = 0.008			F = 2.741; *p*-value = 0.066; η^2^_p_ = 0.02		
	Tot (*N* = 308) 18.5 (22.9)								
Specific Antecedents ***	39.2 (41.6)	41.5 (41)	45.5 (42.6)	41.2 (43.7)	44.5 (37.6)	51.4 (37.7)	42.3 (42.1)	39.3 (42.2)	48.9 (40.6)
	F = 0.587; *p*-value = 0.557; η^2^_p_ = 0.004			F = 0.659; *p*-value = 0.518; η^2^_p_ = 0.004			F = 1.607; *p*-value = 0.202; η^2^_p_ = 0.01		
	Tot (*N* = 308) 42.7 (41.8)								

* Min and max scores: −195; +195. ** Min and max scores: −70; +70. *** Min and max scores: −125; +125.

**Table 3:** Parental distress descriptive data and ANOVA analyses for educational level.

	Level of Education M (SD)				
	Primary and Middle School (*N* = 20)	Professional School (*N* = 24)	High School (*N* = 94)	Degree (*N* = 122)	PhD/ Specialization (*N* = 48)
TOT BR^2^ *	51.8 (61.4)	77.4 (55.9)	59 (56.6)	65.3 (63.6)	51.7 (70.2)
	F = 0.969; *p*-value = 0.425; η^2^_p_ = 0.01				
	Tot (*N* = 308) 61.3 (61.9)				
Common Antecedents **	14.1 (19.4)	22.2 (19.8)	17.3 (22.1)	20.5 (23.2)	16 (26.6)
	F = 0.769; *p*-value = 0.546; η^2^_p_ = 0.01				
	Tot (*N* = 308) 18.5 (22.9)				
Specific Antecedents ***	37.7 (43.9)	55.1 (39.5)	41.7 (38.6)	44.8 (42.8)	35.6 (45.4)
	F = 1.035; *p*-value = 0.389; η^2^_p_ = 0.01				
	Tot (*N* = 308) 42.7 (41.8)				

* Min and max scores: −195; +195. ** Min and max scores: −70; +70. *** Min and max scores: −125; +125.

**Table 4:** Parental distress descriptive data for job conditions.

	Job Condition M (SD)							
	Housewives (*N* = 33)	Student (*N* = 11)	Government Employee (*N* = 108)	Manager (*N* = 15)	Free-Lancer (*N* = 59)	Teacher (*N* = 44)	Unemployed (*N* = 14)	Other (*N* = 24)
TOT BR^2^ *	46.6 (63.6)	32.2 (76.6)	62.3 (58.6)	73.3 (66)	53.2 (66.9)	85 (49.5)	60.7 (76.5)	59.8 (56.1)
	F = 1.795; *p*-value = 0.088; η^2^_p_ = 0.04							
	Tot (*N* = 308) 61.3 (61.9)							
Common Antecedents **	12.6 (23.7)	8.5 (26)	18.6 (22.3)	24.1 (23.1)	16.7 (24.5)	27.2 (18.5)	14.5 (25.8)	18.5 (21.5)
	F = 1.789; *p*-value = 0.089; η^2^_p_ = 0.04							
	Tot (*N* = 308) 18.5 (22.9)							
Specific Antecedents ***	33.9 (43.9)	23.7 (53.2)	43.7 (39.3)	49.2 (44.6)	36.4 (45)	57.8 (33.5)	46.2 (52.8)	41.3 (37.2)
	F = 1.636; *p*-value = 0.125; η^2^_p_ = 0.04							
	Tot (*N* = 308) 42.7 (41.8)							

* Min and max scores: −195; +195. ** Min and max scores: −70; +70 *** Min and max scores: −125; +125.

**Table 5:** Parental distress descriptive data for couples’ conditions.

	Couples’ Conditions M (SD)		
	Married (*N* = 260)	Cohabiting (*N* = 23)	Separated (*N* = 22)
TOT BR^2^ *	62.8 (59.6)	71 (52.8)	41.3 (86)
	F = 1.521; *p*-value = 0.22; η^2^_p_ = 0.01 Tot (*N* = 308) 61.3 (61.9)		
Common Antecedents **	18.7 (22.5)	22.4 (20.1)	14.3 (29.5)
	F = 0.706; *p*-value = 0.495; η^2^_p_ = 0.005 Tot (*N* = 308) 18.5 (22.9)		
Specific Antecedents ***	44.1 (39.9)	48.6 (36.7)	27 (57.9)
	F = 1.958; *p*-value = 0.143; η^2^_p_ = 0.01 Tot (*N* = 308) 42.7 (41.8)		

* Min and max scores: −195; +195. ** Min and max scores: −70; +70. *** Min and max scores: −125; +125.

**Table 6:** Parental distress descriptive data and ANOVA analyses for work regimen during the first lockdown and child gender.

	Work Regimen during the First Lockdown for COVID-19 * M (SD)					Child’s Gender M (SD)	
	Smart Working (*N* = 140)	Work at the Workplace (*N* = 64)	Suspension of Activities (*N* = 39)	Layoffs (*N* = 12)	Lost Job (*N* = 10)	Male (*N* = 178)	Female (*N* = 130)
TOT BR^2^ *	59.9 (61.1)	56.8 (59.4)	63.7 (69.7)	72.2 (52.1)	75.8 (74.1)	59 (64.6)	64.5 (58.2)
	F = 0.342; *p*-value = 0.850; η^2^_p_ = 0.005					F = 0.604; *p*-value = 0.438; η^2^_p_ = 0.002	
	Tot (*N* = 265) 60.6 (61.9)					Tot (*N* = 308) 61.3 (61.9)	
Common Antecedents **	18.7 (22.2)	16.3 (22.5)	19.2 (25.4)	24.6 (19.3)	18.1 (25)	17.2 (23.8)	20.4 (21.5)
	F = 0.374; *p*-value = 0.827; η^2^_p_ = 0.006					F = 1.506; *p*-value = 0.221; η^2^_p_ = 0.005	
	Tot (*N* = 265) 18.4 (22.6)					Tot (*N* = 308) 18.5 (22.9)	
Specific Antecedents ***	41.1 (41.6)	40.4 (39.8)	44.5 (46.6)	47.5 (37.8)	57.7 (50.9)	41.8 (43.6)	44.1 (39.3)
	F = 0.462; *p*-value = 0.763; η^2^_p_ = 0.007					F = 0.229; *p*-value = 0.632; η^2^_p_ = 0.001	
	Tot (*N* = 265) 42.4 (42)					Tot (*N* = 308) 42.7 (41.8)	

* Min and max scores: −195; +195. ** Min and max scores: −70; +70. *** Min and max scores: −125; +125.

**Table 7:** EF descriptive data and ANOVA analysis for child and parental age.

	Child Age M (SD)			Parental Age M (SD)		
	4–6 (*N* = 70)	7–10 (*N* = 101)	11–13 (*N* = 137)	<36 (*N* = 50)	36–45 (*N* = 163)	>45 (*N* = 95)
EF Working Memory	11.2 (3.7)	12.3 (3.2)	12.6 (3.4)	11.3 (4)	12.3 (3.2)	12.4 (3.3)
	F = 3.491; *p*-value = 0.032; η^2^_p_ = 0.02			F = 2.098; *p*-value = 0.124; η^2^_p_ = 0.01		
	Tot (*N* = 308) 12.2 (3.4)					
EF Attentional Control	11.5 (2.9)	11.8 (2.6)	12.1 (3.2)	11.5 (3.2)	12 (2.8)	11.9 (3.2)
	F = 1.184; *p*-value = 0.308; η^2^_p_ = 0.008			F = 0.571; *p*-value = 0.566¸ η^2^_p_ = 0.004		
	Tot (*N* = 308) 11.9 (3)					
EF Planning	10.2 (3.3)	11 (2.6)	11.7 (3.2)	10.3 (3.3)	11.2 (2.8)	11.3 (3.2)
	F = 5.188; *p*-value = 0.006; η^2^_p_ = 0.03			F = 2.019; *p*-value = 0.135; η^2^_p_ = 0.01		
	Tot (*N* = 308) 11.1 (3.1)					
EF Shifting	11.4 (3.5)	12 (3.1)	12.4 (3.3)	11.2 (3.8)	12.3 (3.1)	12.1 (3.3)
	F = 2.192; *p*-value = 0.113; η^2^_p_ = 0.01			F = 2.023; *p*-value = 0.134; η^2^_p_ = 0.01		
	Tot (*N* = 308) 12 (3.3)					
EF Inhibition	10.8 (3.1)	12 (3)	12.6 (3.6)	10.8 (3.7)	12.3 (3.1)	12 (3.6)
	F = 5.926; *p*-value = 0.001; η^2^_p_ = 0.04			F = 3.873; *p*-value = 0.022; η^2^_p_ = 0.02		
	Tot (*N* = 308) 12 (3.4)					
EF Tot	55.2 (14.8)	59.2 (12.6)	61.5 (15.4)	55.2 (16.2)	60.3 (13.4)	59.9 (15.3)
	F = 4.387; *p*-value = 0.013; η^2^_p_ = 0.03			F = 2.465; *p*-value = 0.087; η^2^_p_ = 0.01		
	Tot (*N* = 308) 59.3 (14.6)					

Min and max scores for each subscale: 4; 16.

**Table 8:** EF descriptive data for educational level.

	Educational Level M (SD)				
	Primary and Middle School (*N* = 20)	Professional School (*N* = 24)	High School (*N* = 94)	Degree (*N* = 122)	PhD/ Specialization (*N* = 48)
EF Working Memory	12 (2.8)	12.4 (2.9)	12.1 (3.2)	12.6 (3.3)	11.3 (4.4)
	F = 1.117; *p*-value = 0.348; η^2^_p_ = 0.01				
	Tot (*N* = 308) 12.2 (3.4)				
EF Attentional Control	11.9 (2.8)	12.2 (2)	11.8 (2.6)	12.3 (3)	10.9 (3.8)
	F = 2.040; *p*-value = 0.089; η^2^_p_ = 0.03				
	Tot (*N* = 308) 11.9 (3)				
EF Planning	10.9 (2.3)	11.5 (2.7)	11 (2.7)	11.3 (3.4)	10.6 (3.5)
	F = 0.608; *p*-value = 0.657; η^2^_p_ = 0.008				
	Tot (*N* = 308) 11.1 (3.1)				
EF Shifting	12.7 (2.2)	12.7 (2.5)	12.1 (3.2)	12.2 (3.3)	10.9 (3.9)
	F = 1.927; *p*-value = 0.106; η^2^_p_ = 0.02				
	Tot (*N* = 308) 12 (3.3)				
EF Inhibition	13.3 (2.5)	12.8 (3.2)	11.8 (3.2)	12.2 (3.4)	10.8 (3.8)
	F = 2.646; *p*-value = 0.034; η^2^_p_ = 0.03				
	Tot (*N* = 308) 12 (3.4)				
EF Total	60.9 (10.4)	61.8 (10.9)	59 (12.9)	60.7 (15.1)	54.7 (18.4)
	F = 1.737; *p*-value = 0.142; η^2^_p_ = 0.02				
	Tot (*N* = 308) 59.3 (14.6)				

**Table 9:** EF descriptive data and ANOVA analyses for parental work regimen during the first lockdown, child gender, and child with typical/atypical development condition.

	Work Regimen during the First Lockdown for COVID-19 * M (SD)					Child Gender M (SD)		Child Typ/Atyp Development M (SD)	
	Smart Working (*N* = 140)	Work at the Workplace (*N* = 64)	Suspension of Activities (*N* = 39)	Layoffs (*N* = 12)	Lost Job (*N* = 10)	Male (*N* = 178)	Female (*N* = 130)	No (*N* = 270)	Yes (*N* = 38)
EF Working Memory	12.3 (3.4)	11.6 (3.5)	12.1 (3.4)	12.2 (3.5)	13.3 (3.5)	12.3 (3.4)	11.9 (3.5)	12.3 (3.5)	11.1 (2.8)
	F = 0.706; *p*-value = 0.589; η^2^_p_ = 0.011					F = 0.980; *p*-value = 0.323; η^2^_p_ = 0.003		F = 4.240; *p*-value = 0.040; η^2^_p_ = 0.014	
	Tot (*N* = 265) 12.1 (3.4)					Tot (*N* = 308) 12.2 (3.4)		Tot (*N* = 308) 12.2 (3.4)	
EF Attention Control	12 (3)	11.3 (3)	12.1 (2.8)	13.2 (1.7)	12.7 (3.2)	11.9 (2.8)	11.9 (3.2)	12 (3)	10.8 (2.3)
	F = 1.543; *p*-value = 0.190; η^2^_p_ = 0.023					F = 0.002; *p*-value = 0.969; η^2^_p_ = 0.00		F = 5.271; *p*-value = 0.022; η^2^_p_ = 0.017	
	Tot (*N* = 265) 11.9 (2.9)					Tot (*N* = 308) 11.9 (3)		Tot (*N* = 308) 11.9 (3)	
EF Planning	11.4 (3)	10.5 (2.8)	11.1 (3.2)	11.2 (3.6)	13.1 (2.5)	10.9 (3)	11.3 (3.2)	11.1 (3.1)	10.8 (2.5)
	F = 1.959; *p*-value = 0.101; η^2^_p_ = 0.029					F = 1.078; *p*-value = 0.300; η^2^_p_ = 0.004		F = 0.413; *p*-value = 0.521; η^2^_p_ = 0.001	
	Tot (*N* = 265) 11.2 (3)					Tot (*N* = 308) 11.1 (3.1)		Tot (*N* = 308) 11.1 (3.1)	
EF Shifting	12 (3.2)	11.6 (3.5)	11.7 (3.2)	12.8 (2.8)	13.5 (3.5)	12.1 (3.2)	11.9 (3.4)	12.2 (3.3)	11 (2.7)
	F = 0.918; *p*-value = 0.454; η^2^_p_ = 0.014					F = 0.106; *p*-value = 0.745; η^2^_p_ = 0.00		F = 3.806; *p*-vlaue = 0.052; η^2^_p_ = 0.012	
	Tot (*N*= 265) 12 (3.3)					Tot (*N* = 308) 12 (3.3)		Tot (*N* = 308) 12 (3.3)	
EF Inhibition	12.2 (3.3)	11.4 (3.6)	12 (3.5)	12.7 (2.5)	12.4 (2.8)	11.8 (3.3)	12.2 (3.5)	12 (3.4)	11.8 (3)
	F = 0.737; *p*-value = 0.567; η^2^_p_ = 0.011					F = 0.704; *p*-value = 0.402; η^2^_p_ = 0.002		F = 0.055; *p*-value = 0.816; η^2^_p_ = 0.00	
	Tot (*N* = 265) 12 (3.3)					Tot (*N* = 308) 12 (3.4)		Tot (*N* = 308) 12 (3.4)	
EF Total	60 (14.3)	56.5 (15)	59.1 (14.3)	62.3 (13.1)	65 (14.3)	59.3 (14)	59.4 (15.4)	59.8 (15)	55.8 (10.6)
	F = 1.181; *p*-value = 0.319; η^2^_p_ = 0.018					F = 0.013; *p*-value = 0.908; η^2^_p_ = 0.00		F = 2.551; *p*-value = 0.111; η^2^_p_ = 0.008	
	Tot (*N* = 265) 59.3 (14.5)					Tot (*N* = 308) 59.3 (14.6)		Tot (*N* = 308) 59.3 (14.6)	

Min and max scores for each subscale: 4; 16. * concerns work regimen during the first lockdown, 43 answers are missing.

**Table 10:** EF descriptive data and ANOVA analyses for couples’ conditions and psychological support needs.

	Couples’ Conditions M (SD)			Psychological Support Needs M (SD)	
	Married (*N* = 260)	Cohabiting (*N* = 23)	Separated (*N* = 22)	No (*N* = 206)	Yes (*N* = 102)
EF Working Memory	12.3 (3.3)	11.6 (4.2)	12.5 (3.4)	12.5 (3.5)	11.6 (3.2)
	F = 0.451; *p*-value = 0.637; η^2^_p_ = 0.003			F = 4.687; *p*-value = 0.031; η^2^_p_ = 0.015	
	Tot (*N* = 308) 12.2 (3.4)				
EF Attentional Control	12 (2.8)	11.5 (3.5)	11.5 (3.9)	12.2 (3)	11.3 (2.7)
	F = 0.596; *p*-value = 0.552; η^2^_p_ = 0.004			F = 5.648; *p*-value = 0.018; η^2^_p_ = 0.018	
	Tot (*N* = 308) 11.9 (3)				
EF Planning	11.2 (3)	10 (3.9)	11.2 (2.9)	11.4 (3.4)	10.5 (2.2)
	F = 1.635; *p*-value = 0.197; η^2^_p_ = 0.001			F = 5.509; *p*-value = 0.020; η^2^_p_ = 0.018	
	Tot (*N* = 308) 11.1 (3.1)				
EF Shifting	12.1 (3.2)	12 (3.8)	12 (3.5)	12.3 (3.4)	11.5 (3)
	F = 0.001; *p*-value = 0.999; η^2^_p_ = 0.001			F = 3.951; *p*-value = 0.048; η^2^_p_ = 0.013	
	Tot (*N* = 308) 12 (3.3)				
EF Inhibition	12 (3.3)	12 (3.9)	11.8 (3.9)	12.4 (3.4)	11.1 (3.2)
	F = 0.037; *p*-value = 0.963; η^2^_p_ = 0.001			F = 9.222; *p*-value = 0.003; η^2^_p_ = 0.029	
	Tot (*N* = 308) 12 (3.4)				
EF Tot	59.8 (14.1)	57.3 (18.5)	59.3 (15.3)	60.9 (15.4)	56.2 (12.2)
	F = 0.319; *p*-value = 0.727; η^2^_p_ = 0.002			F = 7.151; *p*-value = 0.008; η^2^_p_ = 0.023	
	Tot (*N* = 308) 59.3 (14.6)				

Min and max scores for each subscale: 4; 16.

**Table 11:** EF descriptive data and ANOVA analyses for order of birth.

	Order of Birth M (SD)		
	First Born (*N* = 209)	Second Born (*N* = 78)	Third Born and beyond (*N* = 21)
EF Working Memory	12.1 (3.6)	12.4 (3.2)	12.5 (2.9)
	F = 0.335; *p*-value = 0.715; η^2^_p_ = 0.002		
	Tot (*N* = 308) 12.2 (3.4)		
EF Attentional Control	11.9 (3)	11.9 (2.8)	11.7 (2.9)
	F = 0.062; *p*-value = 0.940; η^2^_p_ = 0.001		
	Tot (*N* = 308) 11.9 (3)		
EF Planning	11.1 (3.2)	11.1 (2.8)	11.2 (3.3)
	F = 0.033; *p*-value = 0.968; η^2^_p_ = 0.001		
	Tot (*N* = 308) 11.1 (3.1)		
EF Shifting	11.9 (3.4)	12.2 (3.1)	12.2 (2.7)
	F = 0.281; *p*-value = 0.755; η^2^_p_ = 0.002		
	Tot (*N* = 308) 12 (3.3)		
EF Inhibition	11.9 (3.4)	12.2 (3.3)	12.3 (3.3)
	F = 0.301; *p*-value = 0.740; η^2^_p_ = 0.002		
	Tot (*N* = 308) 12.2 (3.4)		
EF Total	59 (15.1)	60 (13.5)	60.1 (13.6)
	F = 0.154; *p*-value = 0.857; η^2^_p_ = 0.001		
	Tot (*N* = 308) 59.3 (14.6)		

Min and max scores for each subscale: 4; 16.

**Table 12:** EF descriptive data and ANOVA analyses for parental job condition.

	Job Condition M (SD)							
	Housewives (*N* = 33)	Student (*N* = 11)	Government Employee (*N* = 108)	Manager (*N* = 15)	Free-Lancer (*N* = 59)	Teacher (*N* = 44)	Unemployed (*N* = 14)	Other (*N* = 24)
EF Working Memory	12.2 (3.6)	12.3 (3.6)	12.2 (3.2)	12.4 (3.6)	12.4 (3.4)	12.1 (3.9)	12.1 (3.3)	11.2 (3.7)
	F = 0.306; *p*-value = 0.951; η^2^_p_ = 0.007							
	Tot (*N* = 308) 12.2 (3.4)							
EF Attention Control	12 (2.9)	11.7 (3.5)	12 (2.4)	11.4 (3.5)	11.8 (3.3)	11.9 (3.8)	11.7 (3.2)	11.8 (2.7)
	F = 0.116; *p*-value = 0.997; η^2^_p_ = 0.003							
	Tot (*N* = 308) 11.9 (3)							
EF Planning	11.2 (2.5)	10.5 (3.6)	11.4 (2.7)	11.6 (2.9)	11.1 (3.1)	11 (3.7)	11.5 (3.3)	9.6 (3.5)
	F = 1.087; *p*-value = 0.371; η^2^_p_ = 0.02							
	Tot (*N* = 308) 11.1 (3.1)							
EF Shifting	12.3 (3.5)	11.3 (2.8)	12.6 (2.9)	12.4 (3.3)	11.9 (3.4)	11.2 (3.8)	11.9 (3.8)	11.2 (3.2)
	F = 1.206; *p*-value = 0.299; η^2^_p_ = 0.03							
	Tot (*N* = 308) 12 (3.3)							
EF Inhibition	12.4 (3.5)	10.4 (2.6)	12.5 (2.7)	12 (3.5)	12 (3.4)	11.7 (4.2)	10.9 (3.7)	10.7 (3.9)
	F = 1.548; *p*-value = 0.151; η^2^_p_ = 0.03							
	Tot (*N* = 308) 12 (3.4)							
EF Total	60.2 (14)	56.4 (14.2)	61 (11.7)	59.8 (15.9)	59.4 (15.7)	58 (18.6)	58.2 (16.1)	54.8 (15.3)
	F = 0.675; *p*-value = 0.693; η^2^_p_ = 0.02							
	Tot (*N* = 308) 59.3 (14.6)							

Min and max scores for each subscale: 4; 16.

**Table 13:** Correlation between BR2 and EF variables.

Variables	1	2	3	4	5	6	7	8	9
1. BR^2^ Tot	-								
2. Common Antecedents	0.920 **	-							
3. Specific Antecedents	0.977 **	0.814 **	-						
4. EF Tot	0.333 **	0.289 **	0.334 **	-					
5. EF Working Memory	0.308 **	0.256 **	0.316 **	0.897 **	-				
6. EF Attention Control	0.294 **	0.247 **	0.299 **	0.906 **	0.839 **	-			
7. EF Planning	0.256 **	0.238 **	0.249 **	0.861 **	0.689 **	0.730 **	-		
8. EF Shifting	0.325 **	0.268 **	0.335 **	0.924 **	0.798 **	0.786 **	0.723 **	-	
9. EF Inhibition	0.299 **	0.280 **	0.289 **	0.877 **	0.668 **	0.706 **	0.720 **	0.810 **	-

** *p* ≤ 0.01.

**Table 14:** Linear regression analysis relating specific antecedents and each component of ESA.

Dependent Variables	Model	R	R Square	R Square Change	F Change	gl1	gl2	Sig. F Change
EF Working memory	1	0.316	0.100	0.097	33.832	1	306	0.000
EF Attention control	1	0.299	0.090	0.087	30.153	1	306	0.000
EF Planning	1	0.249	0.062	0.059	20.232	1	306	0.000
EF Shifting	1	0.335	0.112	0.109	38.642	1	306	0.000
EF Inhibition	1	0.289	0.084	0.081	27.915	1	306	0.000

Predictors: (constant): SPECIFIC ANTECEDENTS.

## Data Availability

The data presented in this study are available upon request from the corresponding author.
